# Effects of Bulk Tank Milk, Waste Milk, and Pasteurized Waste Milk on the Intake, Ruminal Parameters, Blood Parameters, Health, and Performance of Dairy Calves

**DOI:** 10.3390/ani11123552

**Published:** 2021-12-14

**Authors:** Sabrina de Freitas Vieira, Sandra Gesteira Coelho, Hilton do Carmo Diniz Neto, Hemily Cristina Menezes de Sá, Barbara Pironi Pereira, Bianca Souza Ferreira Albuquerque, Fernanda Samarini Machado, Luiz Gustavo Ribeiro Pereira, Thierry Ribeiro Tomich, Isis Rodrigues Toledo Renhe, Mariana Magalhães Campos

**Affiliations:** 1 Departamento de Zootecnia, Escola de Veterinária, Universidade Federal de Minas Gerais(UFMG), Belo Horizonte 30161-970, MG, Brazil; sabrinavieira42@yahoo.com.br (S.d.F.V.); sandragesteiracoelho@gmail.com (S.G.C.); hiltondinizneto@gmail.com (H.d.C.D.N.); hemilly.mg@hotmail.com (H.C.M.d.S.); 2 Embrapa Gado de Leite, Empresa Brasileira de Pesquisa Agropecuária (EMBRAPA), Juiz de Fora 36038-330, MG, Brazil; barbarapironepereira@gmail.com (B.P.P.); biasouzafa@gmail.com (B.S.F.A.); fernanda.machado@embrapa.br (F.S.M.); luiz.gustavo@embrapa.br (L.G.R.P.); thierry.tomich@embrapa.br (T.R.T.); 3 Instituto de Laticínios Cândido Tostes, Empresa de Pesquisa Agropecuária de Minas Gerais (EPAMIG), Juiz de Fora 36045-560, MG, Brazil; isis@epamig.br

**Keywords:** growth, pasteurization, volatile fatty acids, weight gain

## Abstract

**Simple Summary:**

Waste milk (WM) is commonly used in the feeding of calves. Due to its legal prohibition in commercialization, the destination of WM has become an environmental issue for dairy farms. Many dairy farms pasteurize WM, focusing on reducing the microbial load and related sanitary challenges. However, pasteurized milk may still contain toxins of bacterial origin, spores, and antibiotic residues. Few studies have evaluated the effects of whole milk, WM, and pasteurized WM (PWM) on the intake, ruminal parameters, blood parameters, health, and performance of dairy calves. In our study, feeding WM or PWM did not show significant negative effects on the intake, ruminal parameters, blood parameters, health, or performance of dairy calves. Understanding the effects of using WM and PWM on the health and performance of dairy calves requires further investigation.

**Abstract:**

The aim of this study was to evaluate the effects of bulk tank milk (BTM), WM, and PWM on the intake, ruminal parameters, blood parameters, health, and performance of dairy calves. Forty-five male crossbred dairy calves (Gyr × Holstein) were used. On their fourth day of age, animals were grouped according to body weight, serum protein levels, and genetic composition. Three treatments were assessed: BTM (*n* = 15), WM from cows in antibiotic treatment (*n* = 15), and PWM via high-temperature, short-time pasteurization (72–74 °C for 16 s) (*n* = 15). During the experimental period (from 4 to 60 d of age), animals were fed 6 L of milk/d, divided into two equal meals. Water and concentrate were provided *ad libitum*. Daily measurements were made for milk, concentrate, and water intakes, as well as for fecal and respiratory scores. Rumen fluid and blood were sampled weekly. The following parameters were evaluated: volatile fatty acids (VFAs), pH and ammonia-N in rumen fluid, and β-hydroxybutyrate (BHB) and glucose in blood. Animals were weighed at birth, 4 d of age, and weekly up to 60 d of age. At the end of the experimental period (60 ± 1 d), all animals were euthanized for pulmonary evaluation. The randomized complete design with an interaction between treatment and week was the experimental method of choice for testing the hypothesis of the treatment’s effect on all evaluated outcomes. Animals in the BTM treatment had higher milk dry matter intake (DMI), followed by WM and PWM calves. Concentrate DMI was lower for BTM in comparison to WM and PWM calves. However, total DMI showed no significant differences between treatments. The rumen fluid from calves receiving PWM had higher concentrations of acetate and propionate than that of BTM and WM animals. No differences were observed between treatments for blood glucose and BHB concentrations. Health parameters (fecal and respiratory scores) and pneumonia occurrence showed no significant difference between treatments. No differences were observed for average daily gain (ADG) or body growth. Feeding WM and PWM did not show significant negative effects on the intake, ruminal parameters, blood parameters, health, or performance of dairy calves.

## 1. Introduction

Waste milk (WM) is a milk secretion not suitable for commercialization and originates from cows milked immediately after calving (colostrum and transition milk), undergoing treatment with pharmacological products (antibiotics, anti-inflammatory, or antiparasitic drugs) or cows fully treated but still within the withdrawal period [1]. Because it is legally prohibited for commercialization, WM’s destination has become an environmental issue for dairy farms. The appropriate discharge of such substances requires waste treatment systems such as biodigesters and settling ponds.

On the other hand, providing a liquid diet of high nutritional value and low cost for young calves is another challenge in dairy production. Attempting to avoid the obstacles and high costs of correct discharge and reduce feeding expenses, many dairy farms commonly use WM in the feeding of calves.

Although feeding WM is an economically viable option, this practice may pose health risks for calves, given the high microbial load and presence of antibiotic residues in WM [2]. Using WM of cows affected by infections, such as mastitis, in the feeding of calves constitutes another controversial subject [1]. In addition, WM presents variable nutritional composition, mainly due to the presence of transition milk and low-quality colostrum [3].

In this sense, many dairy farms pasteurize WM, focusing on reducing the microbial load and overcoming the sanitary challenges related to such milk. Besides decreasing 99% of pathogenic microorganisms [4], pasteurization causes minimal alterations in the composition of milk, and pasteurized waste milk (PWM) can stimulate weight gain and improve the development of calves [5]. The use of PWM in the feeding of animals may reduce the development of diseases such as diarrhea and pneumonia [6].

However, many different and controversial results have been reported in the scientific literature regarding WM and PWM use. Studies have been done to evaluate the differences in health parameters and performance in calves fed WM and bulk tank milk (BTM), but no differences were observed between the evaluated groups [7,8]. In contrast, in another study, the animals receiving WM had higher concentrate intake and more weight gain than animals fed BTM [1]. When evaluating milk pasteurization, calves that consumed PWM presented better health parameters than those that consumed milk replacer [9,10]. However, a recent study did not find any development or health improvements in calves fed PWM in comparison to WM or BTM [1].

During the milk-feeding of calves, small amounts of milk pass through the rumen. In this sense, the antibiotic residue and the microbial properties of WM and PWM may alter the rumen microbiota, directly affecting the ruminal parameters of animals in these treatments [11,12]. However, this subject is little discussed in the scientific literature.

The aim of this study was to evaluate the effects of BTM, WM, and PWM on the intake, ruminal parameters, blood parameters, health, and performance of dairy calves. Our hypothesis was that although feeding WM does not hinder the growth or development of calves, it may negatively affect their health. Therefore, pasteurization may be a valid strategy in avoiding these negative consequences.

## 2. Materials and Methods

The experiment was conducted at the Embrapa Gado de Leite Experimental Farm, located in Coronel Pacheco, Minas Gerais, Brazil. During the trial period, the average temperature was 27 °C (maximum 37.4 °C, minimum 8.2 °C). The mean relative humidity during the study period was 72% (maximum 85 °C, minimum 40 °C). Procedures were approved by the Ethics Committee on Animal Use of Embrapa Dairy Cattle (CEUA number: 9849040419).

### 2.1. Calves, Housing, Management, and Treatments

Forty-five male Holstein × Gyr crossbred dairy heifers, with genetic composition of 5/8 or more Holstein and 3/8 or less Gyr were used. After birth, newborn animals were immediately separated from their dams and had their umbilical cord immersed in iodine solution (10%) for 3 consecutive days. Before completing 6 h of age, animals were weighed and received 10% of their body weight in colostrum with Brix = 25% (79.8 g/L of IgG) [13]. Colostrum with a result Brix < 25 was densified using a colostrum replacer (Saskatoon Colostrum Company, Saskatoon, Canada; DM = 67.1) until reaching a Brix value of 25%.

In the first 3 days, calves were housed in individual suspended cages (1.50 m × 0.80 m; Intergado Ltd.a, Contagem, Brazil) with hay beds. Blood samples (5 mL) for the assessment of passive immunity transfer were obtained 48 h after the initial colostrum intake. Blood samples were collected via jugular venipuncture with a clot activator tube (Labor Import, Osasco, Brazil), centrifuged at 1800× *g* for 10 min at room temperature (22–25 °C), to measure total serum protein using a refractometer (Serum protein REF-301, Biocotek, Ningbo, China). Male calves with low serum protein (<5.5) were not enrolled in the present study [14]. There were no differences (*p* = 0.38) between groups for passive immune transfer, with average and standard deviations for total serum protein of 6.6 ± 0.81, 6.95 ± 0.88, and 6.63 ± 0.65 g/dL for calves in the BTM, WM, and PWM groups, respectively.

At 2 to 3 d of age, heifers were fed 6 L/d of transition milk divided into two equal meals (0800 and 1600 h) offered in buckets provided with rubber teats (Milkbar^®^, Waipu, New Zealand). Water was provided ad libitum from day one.

On their fourth d of age, calves were randomly distributed according to body weight, serum protein levels, and genetic composition into three treatments: BTM (*n* = 15), WM from cows in antimicrobial treatments (*n* = 15) (cows with clinical mastitis, placental retention, metritis, or foot infections), and PWM (*n* = 15), pasteurized by high-temperature, short-time pasteurization (72–74 °C for 16 s) with a plate pasteurizer (West, Juiz de Fora, Brazil).

During the experimental period (4 to 60 d of age), animals were housed in individual sand-bedded pens (1.25 × 1.75 m, tethered with 1.2-m-long chains). All experimental groups received 6 L/d of milk, divided into two meals (BTM = 0900 and 1500; WM = 1000 and 1600; and PWM = 1100 and 1700). The milk from each treatment was homogenized with the aid of a ladle, always before the meal. Experimental treatments were fed in buckets provided with rubber teats (Milkbar^®^, New Zealand). A solid diet was offered ad libitum, starting at the fourth day of age (10% orts of solid feed). The diet was comprised of ground corn, soybean meal, and mineral and vitamin supplements (Prima/DSM, São Paulo, Brazil) (Table 1).

Milk pasteurization was performed daily (1000 h) with a plate pasteurizer (West, Juiz de Fora, Brazil). After homogenization of WM, the daily volume required for feeding PWM calves was pasteurized. The presence of alkaline phosphatase and peroxidase was assessed daily with reagent strips (Cab-Lab, São Paulo, Brazil) to verify the efficiency of the pasteurization process. The PWM was only used after certification of the absence of alkaline phosphatase and presence of peroxidase. Calves in the PWM treatment had their first meal immediately after pasteurization (milk with temperature of 38 °C), and the remaining milk was refrigerated for 6 h (4 °C) until the time of the second meal. At that time, PWM was heated to 38 °C and fed to calves.

### 2.2. Intake

Feed intake (milk, starter, and water) was measured daily by the difference between the supplied and orts. Starter and water were weighed on 5-g precision scales (Prix 3 Plus, Toledo^®^, São Bernardo do Campo, São Paulo, Brazil).

Dry matter intake (DMI) was calculated for milk and starter, considering their respective dry matter (DM) content. Total DMI was calculated from the sum of the consumed quantities of each supplied feed (MS of the milk + starter).

Gross energy intake (GEI) was calculated as the difference between the gross energy (GE) of the supplied, consumed, and leftover feeds (milk + starter). Milk GE was calculated based on the calculation: GE (Mcal/kg milk) = (0.0911 × % fat) + (0.0586 × % protein) + (0.0395 × % lactose) [15].

### 2.3. Nutrient Composition Analysis

The supplied starter was sampled weekly. Samples were stored at −20 °C until analysis. Analysis began by oven-drying samples for 72 h at 55 °C. Then, samples were ground in a Wiley mill (Model 3, Arthur H. Thomas Co., Philadelphia, PA, USA) to pass a 1-mm sieve. Crude protein (CP) (988.05 AOAC method), ether extract (EE) (920.9 AOAC method), ashes (942.05 AOAC method), neutral detergent fiber (NDF), and acid detergent fiber (ADF) were quantified [16].

Milk samples for each treatment were obtained prior to feeding at both mealtimes (morning and afternoon). For the milk composition analysis (crude protein, fat content, lactose, and total dry extract) and somatic cell count (SCC), samples were stored in flasks containing Bronopol^®^ (Prolab, São Paulo, Brazil). For the total bacteria count (TBC) analysis, milk samples were stored in flasks containing Azidiol (Prolab, São Paulo, Brazil).

Flow cytometry analyses were performed to assess the concentration of SCC and TBC in milk samples (Bentley 2300 Combi & Bactocount IBC, Laboratory of Milk Quality of Embrapa Dairy Cattle, Juiz de Fora, Brazil). To evaluate the efficiency of the pasteurization process, pre-pasteurization and immediately after pasteurization, milk samples were collected in flasks containing Azidiol (Prolab, São Paulo, Brazil).

Organic matter was incinerated at 550 °C to determine the ash content (ISO 936: 1998). Nitrogen content was determined by the Micro Kjeldahl method (FIL 20B: 1993), with a 6.35 factor for the conversion of crude protein and casein and a 3.6 factor for non-protein nitrogen.

### 2.4. Rumen Variables and Analyses

Rumen fluid was obtained weekly, 4 h after the morning meal of each treatment. Sampling was performed with an oroesophageal tube. Samples were filtered twice and had their pH measured (T-1000 pH Meter, Tekna, Araucária, Brazil).

For the determination of ammonia-N and volatile fatty acid (VFA) concentrations, 5 and 10 mL of rumen fluid were filtered and acidified with 1 mL of sulfuric acid (500 mL/L) and 1 mL of metaphosphoric acid (20%), respectively. Samples were stored at −20 °C until analysis.

Ammonia-N was quantified by the colorimetric method [17]. After the Kjeldahl test, absorbance was measured at 630 nm (Thermo Fisher Scientific, Madison, WI, USA) with magnesium oxide and calcium chloride according to the 920.03 method (AOAC, 1990). Samples for the VFA analysis were evaluated by high-performance liquid chromatography (Waters Alliance e2695 Chromatograph, Waters Technologies do Brasil LTDA, Barueri, São Paulo, Brazil).

### 2.5. Blood Variables and Analyses

Blood samples were obtained by jugular venipuncture after local asepsis with 70% alcohol and were used for β-hydroxybutyrate (BHB) and glucose analyses. Sampling was performed weekly, 3 h after the first meal of each treatment.

For the BHB analysis, tubes without anticoagulant were used, whereas tubes for the glucose analysis contained sodium fluoride (Vacutainer; Becton, Dickinson and Company, São Paulo, Brazil). Samples were immediately transported on ice to the laboratory and were centrifuged at 3000× *g* for 10 min at room temperature (22–25 °C). Duplicates were made for serum and plasma aliquots and stored at −20 °C for later analysis.

BHB and glucose serum concentrations were determined with an auto-analyzer (Cobas Mira Plus, Roche Diagnostic Systems, Risch-Rotkreuz, Switzerland) using commercial kits for BHB (Ranbut-D-3-Hidroxibutyrate, Randox Laboratories Ltd., Antrim, UK) and glucose (Glucose, Doles, Goiás, Brazil).

### 2.6. Health Parameters

Rectal temperature was measured daily at 0600 h with the aid of a digital thermometer (Ombo Electronics, iColor^®^, G-Tech model, Shenzhen, China) with a measurement range of 32.0 to 43.9 °C. Animals with temperatures higher than or equal to 39.4 °C were classified with hyperthermia.

Fecal score was determined daily, according to the following scores: 0, normal (firm but not hard); 1, soft (does not hold form, piles but spreads slightly); 2, runny (spreads readily to about 6-mm depth); and 3, watery (liquid consistency, splatters) [18]. Fecal score ≥2 was classified as diarrhea and severe diarrhea if the score was equal to 3. All animals with diarrhea, during the days they presented symptoms, received oral fluid therapy twice a day (10 g NaCl, 12 g sodium acetate, 2 g KCl, and 40 g glucose for 2 L water). If hyperthermic, animals received anti-inflammatory drugs for 3 days (flunixin meglumine, JA Saúde Animal, São Paulo, Brazil, 1 mL/45 kg). If hyperthermia persisted for 2 consecutive days, animals received parenteral antibiotic therapy for 5 days (enrofloxacin, Bayer, São Paulo, Brazil, 1 mL/40 kg).

Respiratory disease assessment was performed daily based on rectal temperature and nasal discharge scores, where: 0, normal, serous discharge; 1, small amount of unilateral, cloudy discharge; 2, bilateral, cloudy, or excessive mucus discharge; and 3, copious, bilateral mucopurulent nasal discharge [14]. Animals with pneumonia received anti-inflammatory drugs for 3 days (flunixin meglumine, JA Animal Health, São Paulo, Brazil, 1 mL/45 kg) and parenteral antibiotic therapy for 5 days (enrofloxacin, Bayer, São Paulo, Brazil, 1 mL/40 kg).

### 2.7. Pulmonary Consolidation

At the end of the experimental period (60 ± 1 d), all animals were euthanized following the procedures recommended by the Brazilian National Council of Veterinary Medicine. After euthanasia, the chest cavity was opened, and lungs were removed to assess the occurrence of respiratory disease. The area of lung consolidation was measured based on the score, where: 0 = normal or area of consolidation <1 cm^2^ and 1 = area of consolidation ≥1 cm^2^ [19].

### 2.8. Performance, Feed Efficiency, and Body Measurements

Weight and body measurements were performed at the fourth and seventh days and weekly thereafter. All measurements were made prior to the first meal. Feeding efficiency (FE) was calculated by dividing the mean of average daily gain (ADG) by the total DMI (milk + starter). Animals were weighed in mechanical scales (COIMMA S16.742, Dracena, São Paulo, Brazil). Wither height (WH, distance from the base of the front feet to the withers) and rump height (RH, distance from the base of the rear feet to the rump) were measured using Teletape (Ketchum Deluxe Livestock Measure). The heart girth (HG, circumference of the chest) and rump width (RW, measured immediately and caudally to the front limbs) were measured with a measuring tape (38.68.150.000, Vonder, Curitiba, Brazil). Measurements were performed on plain ground, allowing animals to stand with limbs positioned symmetrically in relation to the floor.

### 2.9. Statistical Analysis

Data were analyzed using R software (R Core Team, 2019–version 4.1.2). Sample size calculations using a power of 80% and a significance level of 0.05 indicate that a difference in most of the parameters would be evident with 15 calves per group. The continuous outcomes such as intake (milk, starter, total DMI, total GE, CP intake, and water intake), performance, feed efficiency, body measurements, blood, and ruminal parameters were analyzed using a linear mixed-effect model (nlme package). Treatment, genetic composition, day/week, and interaction were included as factors for fixed effects and animals as random effects. Birth weight and total serum protein were tested as covariates and included in the model only if significant (*p* < 0.05). All models were verified graphically for normality and homoscedasticity of residuals and tested with the Shapiro–Wilk and Bartlett tests. A 95% confidence interval was adopted to verify the null hypothesis, and *p*-values were produced with a Tukey test.

Variables such as initial and final BW, total weight gain, and passive immunity transfer were analyzed using a linear mixed-effect model (nlme package). Treatment and genetic composition were included as fixed effects and animals as random effects. Variables of milk composition (DM, fat content, crude protein, casein, non-protein nitrogen, lactose, and minerals) and quality (SCC and TBC) were analyzed using a linear mixed-effect model (nlme package). Treatment was included as fixed effects and samples as random effects. The SCC and TBC data were log-transformed (log10) prior to the analysis.

The categorical outcomes, fecal and respiratory scores, were analyzed using a non-parametric aligned rank transformation test, implemented in the R package ARTool. A 95% Confidence Interval was also adopted for the non-parametric tests. The occurrence of diarrhea and respiratory diseases (area of consolidation) were analyzed with a chi-squared test (stats package). For all analyses, a *p*-value of <0.05 was considered statistically significant.

## 3. Results

### 3.1. Milk Composition and Efficiency of Pasteurization

Milk fat content was higher for the BTM (4.24%) and WM (4.10%) treatments in comparison to the PWM treatment (3.76%) (*p* < 0.05) (Table 1). WM and PWM had higher crude protein content (3.46 and 3.49%, respectively) than BTM (3.30%; *p* < 0.05). No significant difference was observed for the percentage of casein in the BTM, WM, and PWM treatments (2.71, 2.64, 2.53%, respectively; *p* = 0.22). However, WM and PWM had a higher content of non-protein nitrogen (0.13 and 0.12%, respectively) than BTM (0.10%; *p* < 0.05). The lactose concentration was significantly higher in the BTM treatment (4.46%) than in the WM and PWM treatments (4.33%; *p* < 0.05).

BTM had a significantly lower concentration of minerals (0.69%; *p* < 0.05) than WM and PWM (0.72 and 0.73%, respectively). BTM had the highest DM content (13.04%), followed by WM (12.82%) and PWM (12.48%; *p* < 0.05). WM had a higher SCC (1740.15 × 10^3^ cells/mL) than BTM and PWM (366.81 and 1424.67 × 10^3^ cells/mL, respectively; *p* < 0.05). WM had a higher TBC (548.37 × 10^3^ CFU/mL) than BTM and PWM (19.79 and 295.41 × 10^3^ CFU/mL, respectively; *p* < 0.05).

Milk samples presented a TBC of 544 × 10^3^ CFU/mL prior to the pasteurization and 195 × 10^3^ CFU/mL immediately after the pasteurization process, resulting in a 64.15% reduction in the microbial load of the WM.

### 3.2. Intake

Milk intake was higher in the BTM treatment (772.37 g DM/d; Table 2), followed by WM (764.29 g DM/d) and PWM (740.87; *p* < 0.05).

**Figure 1:**
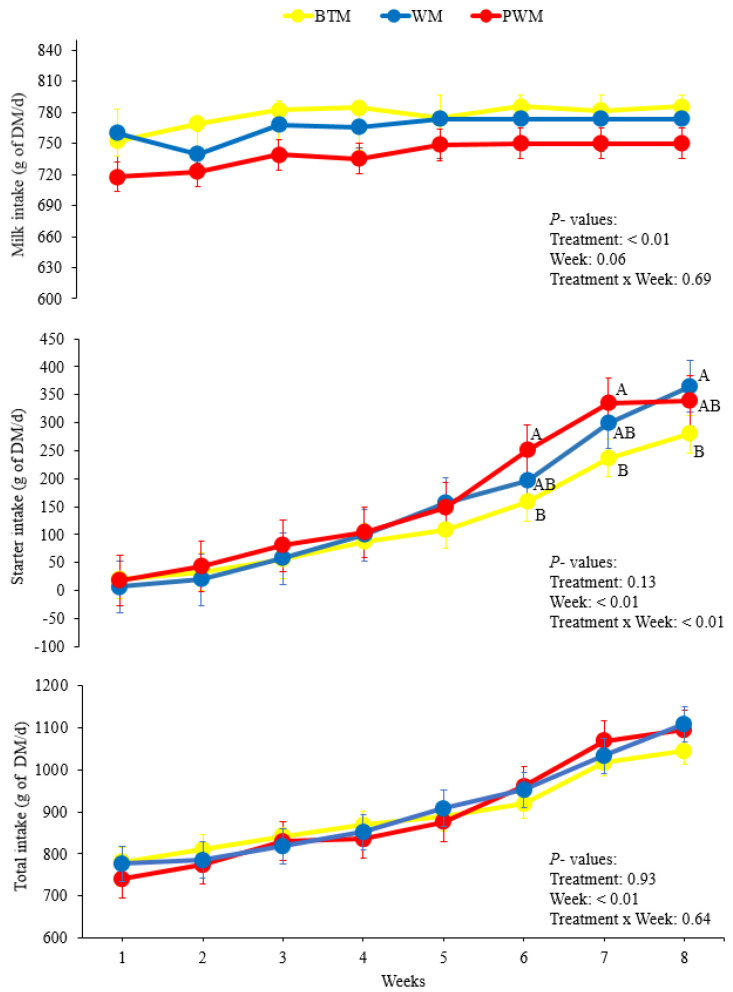
Dry matter intake (milk, starter, and total) of dairy calves fed bulk tank milk (BTM, *n* = 15), waste milk (WM, *n* = 15), and pasteurized waste milk (PWM, *n* = 15) during the period from 4 to 60 d. Bars represent SEM. Letters represent statistical difference between treatments (*p* < 0.05).

Starter intake was significantly affected by week and week × treatment interaction (*p* < 0.05; Table 2). No difference regarding starter intake was found between treatments from weeks 1 to 5 (Figure 1; *p* > 0.05). However, during weeks 6 and 7, calves fed BTM had a lower starter intake in comparison to PWM (*p* < 0.05) and WM animals (Figure 1). During week 8, a similar pattern was found: Calves receiving BTM had a lower starter intake (280.03 g DM/d) than WM, whereas WM (364.73 g DM/d) had similar values to PWM (339.73 g DM/d) (*p* > 0.05) (Figure 1). Regarding total DMI, only the week effect was significant (*p* < 0.01; Figure 1), with a gradual increase each week in total DMI.

Gross energy was significantly affected by week and week × treatment interaction (*p* < 0.01; Table 2). During weeks 6 and 7, the PWM treatment showed a higher GE intake (5.41 and 7.91 Mcal/d for weeks 6 and 7, respectively) in comparison to the BTM treatment (4.0 and 6.25 Mcal/d; *p* < 0.05) but was similar to WM (4.84 and 6.86 Mcal/d; *p* > 0.05). During week 8, both WM and PWM treatments had a higher GE intake (8.76 and 8.83 Mcal/d, respectively) than the BTM treatment (7.03 Mcal/d; *p* < 0.05). A significant effect for the week factor and a significant interaction between week and treatment were observed for the CP values (*p* < 0.01; Table 2). The PWM treatment (69.01 kg/d), from weeks 5 to 7, had a CP intake similar to the WM treatment (65.71 kg/d; *p* > 0.05) and a significantly higher CP intake than the BTM treatment (57.33 kg/d; *p* < 0.05). At week 8, both WM and PWM treatments had a higher CP intake (96.12 and 96.70 kg/d, respectively) than the BTM treatment (79.61 kg/d; *p* < 0.05).

Water intake was significantly affected by week (*p* < 0.01; Table 2). Water intake increased gradually from weeks 1 to 5 (*p* < 0.05) and stabilized from weeks 6 to 8 (*p* > 0.05).

### 3.3. Rumen and Blood Parameters

Only the concentrations of acetate (*p* = 0.01) and propionate (*p* = 0.03) showed significant effects from the treatment. The rumen fluid of calves receiving PWM had higher concentrations of acetate and propionate than that of calves in the BTM and WM treatments (38.48, 33.02, and 32.89 mmol/L acetate and 26.21, 21.22, and 21.68 mmol/L propionate for PWM, BTM, and WM, respectively) (Table 3). The week factor affected acetate, propionate, butyrate, the acetate:propionate ratio, and pH (*p* < 0.05; Table 3). No significant differences were found between treatment for the ammonia-N (*p* > 0.05).

Glucose and BHB concentrations were neither affected by the treatment nor by the interaction between treatment and week (*p* > 0.05; Table 3). However, a significant effect of week was observed for BHB, where lower concentrations were found from weeks 1 to 3 (0.036 mmol/dL) in comparison to weeks 4 to 8 (0.13 mmol/dL; *p* < 0.01; Table 3).

### 3.4. Health Parameters

Fecal score analysis showed a significant effect of week (*p* < 0.0001; Table 4), with higher values observed in week 2 (0.81; *p* < 0.05). No significant differences were observed between the BTM, WM, and PWM treatments (Table 4) for the number of animals with diarrhea (9, 6, and 7 animals, respectively; *p* = 0.72), days with diarrhea (8.21, 5.93, and 5.21 d, respectively; *p* = 0.29), days with fever (0.67, 1.13, and 1.81, respectively; *p* = 0.79), or calves with area of lung consolidation ≥ 1 (3, 7, and 5 calves, respectively; *p* = 0.30).

### 3.5. Performance, Feed Efficiency, and Body Development

Average daily gain was not affected by any of the three treatments (*p* > 0.05). However, ADG was significantly different between weeks, with the second week showing smaller values of ADG (0.44 kg/d, *p* < 0.01; Table 2) No significant differences between BTM, WM, and PWM calves were found for final BW (76.03, 77.43, and 74.09 kg, respectively; *p* > 0.05). Feed efficiency was significantly affected by treatment, week, and treatment x week. BTM and WM calves showed higher FE (0.76 and 0.79, respectively) than PWM animals (0.71; *p* < 0.05). Calves receiving BTM, during weeks 2 and 3, had FE values (0.75) similar to WM animals (0.73; *p* > 0.05) and a significantly higher FE than PWM-fed calves (0.65; *p* < 0.05). For weeks 4 and 7, BTM and WM animals had higher GE values (0.75 and 0.81, respectively) than PWM calves (0.62; *p* < 0.05). In week 6, calves fed PWM had FE values (0.83) similar to animals receiving WM (0.76; *p* > 0.05) and a significantly higher FE than BTM fed calves (0.72; *p* < 0.05).

The week factor significantly affected the body measurement parameters of WH, RH, and RW (*p* < 0.05; Table 2). Values of WH (84.41, 84.15, and 84.28 cm for BTM, WM, and PWM, respectively), RH (87.19, 87.35, and 87.36 cm for BTM, WM, and PWM, respectively), and RW (24.95, 25.01, and 24.82 cm for BTM, WM, and PWM, respectively) gradually increased in each of the weeks of the experiment. The HG parameter was significantly affected both by week and by the interaction between treatment and week. In week 5, calves fed BTM had CG values (89.42 cm) similar to WM animals (87.66 cm; *p* > 0.05), whereas these values were significantly higher than the PWM treatment (87.05; *p* < 0.05).

## 4. Discussion

To our knowledge, this study was the first to simultaneously assess the effects of feeding bulk tank milk (BTM), waste milk (WM), and pasteurized waste milk (PWM) on the intake, ruminal parameters, blood parameters, health, and performance of dairy calves.

Regarding the milk composition, the lower percentage of lactose in WM and PWM treatment and higher values of TBC in the WM treatment were due to the presence of transition milk. Although the WM used in the experiment was obtained only from cows undergoing antimicrobial treatment (for clinical mastitis, placental retention, metritis, or foot infections), some conditions such as placental retention and metritis occurred a few days after calving, while the milk was still considered in a transition stage. In comparison to whole milk, transition milk has higher total solid, fat, and protein contents but lower lactose concentrations [20].

After the homogenization of WM, the fraction of milk required for both meals of calves in the PWM treatment was separated, pasteurized, and stored. This process was performed once per day, with the WM milked in the morning. Even though heating during pasteurization deactivates a large part of lipases, some might have remained active during the storage process, causing the lower fat content observed in PWM. In addition, the elevated SCC for the PWM treatment (1424.67 × 103 cells/mL) may have contributed to an increase in lipase concentrations, further intensifying the lipolysis during the storage period (6 h) and decreasing the fat content in the PWM.

A recent study did not find any differences in the protein contents of BTM, WM, and PWM [1]. In another analysis, while evaluating the nutritional value of WM and PWM, it was not possible to find significant differences in protein (3.51%), fat (3.90%), or lactose (4.42%) contents [21]. In general, many different compositions of WM and PWM are found in the scientific literature [1,3,21]. These differences in composition and nutrient content are mainly related to the differences in volumes of colostrum, transition milk, and milk from cows with clinical mastitis used in the WM.

Regarding the microbial assays, a reduction of 64% in the TBC was observed immediately after pasteurization. Some studies have reported reductions of approximately 98–99% of the microbial load [10,22,23]. Other studies have reported a wider range of reductions, from 20% to 70% [24,25]. Variations in the efficiency of the pasteurization process are related to factors such as temperature, sanitation of the pasteurizer, sanitation during the pasteurization process, and training of the operator. Furthermore, because the pasteurization is not 100% efficient, the microbial quality of the milk before pasteurization is an important factor.

Even though a 64% reduction in TBC was observed directly after pasteurization, the samples obtained immediately before the second meal of animals showed reductions of TBC of 49%, indicating possible recontamination after the pasteurization process. Recontamination of pasteurized milk is common in dairy farms, mainly due to failures in the refrigeration process or a lack of sanitation in equipment and utensils [21,22,25]. Reductions in microbial load in WM from 64.71 CFU/mL to 5.87 CFU/mL have been found after pasteurization [22]. However, in this same study, recontamination also occurred, with microbial load values reaching 30.44 CFU/mL.

The higher milk DMI of calves fed BTM is related to the differences in the nutritional value of the milk used in each treatment. Even though every animal received the same volume of milk every day (6 L), the DM content presented daily fluctuations. Milk used in the BTM treatment presented higher values of DM in comparison to WM and PWM, explaining the higher DMI observed for BTM.

The higher concentrate intakes observed for calves fed WM and PWM from the sixth to eighth week are possibly due to physiological mechanisms of intake regulation. To meet their nutritional demands, animals in the WM and PWM treatments increased their concentrate intake because the DMI obtained from milk was insufficient. Contrary to our findings, in another study, no differences were observed for total milk intake or total concentrate intake between calves receiving WM, PWM, BTM, and pasteurized whole milk [25]. However, the assessment of the milk DMI and other intake parameters was not performed weekly, making full comparisons with our results difficult.

For the ruminal parameters, the higher values of acetate and propionate in the rumen fluid of calves fed PWM are related to the higher intake of concentrate. The gradual increase on each week of the acetate, propionate, and butyrate concentrations, as well as the AC:PRO ratio, are consistent with the increase in solid intake [26].

During the milk-feeding of calves, small amounts of milk pass through the rumen [27,28]. In this sense, the antibiotic residue and the microbial properties of WM and PWM may alter the rumen microbiota, directly affecting the ruminal parameters of animals in these treatments [11,12]. This subject is still not well discussed in the literature and, to the best of our knowledge, only one study has assessed the effect of WM on ruminal parameters. The ruminal parameters of calves fed WM, BTM, and milk replacer have been previously evaluated; however, significant changes were observed only for the concentration of VFA, with higher values of VFA for calves fed milk replacer [12]. The rumen fluid of calves fed WM presented higher concentrations of isovalerate. Because this substance originated from leucine, the authors state that the higher concentrations of isovalerate in the rumen are related to fermentation of milk with elevated protein content.

No differences were observed between treatment for pH, but pH values were reduced from the second to the eighth week. In accordance with our results, the most significant pH changes occur when animals have a higher intake of solid feed, typically between the fourth and seventh week [29].

In the first weeks of life, calves use glucose as a primary source of energy. As the rumen develops, the concentration of glucose decreases and those of VFA progressively increase. After weaning, calves complete their rumen development and VFA produced by ruminal microbiota becomes the primary energy source, justifying BHB concentration increase and glucose concentration decrease [15]. In our study, no glucose changes were found, possibly due to the supply of 6 L of milk/d throughout the period (60 d). Concentrations of BHB were lower in the first and second weeks, in comparison to the other weeks of the experiment, with the increase in BHB starting at the third week related to the steady increase in concentrate intake.

Feeding WM to calves raises many concerns due to the high microbial load the milk may contain, possibly increasing the spread of diseases. This high microbial load may be originated from infections in the mammary gland, ineffective sanitation practices, and inappropriate storage of WM [3]. Pasteurization has arisen as a possible tool for controlling the microbial contamination of milk and reducing the adverse effects on animal health. No relationship between the usage of WM and health parameters was found in our study. Even though the TBC of WM and PWM were above the recommended values [10] (TBC < 20.000 CFU/mL), and the TBC of BTM was below the recommended value, no differences were found in the assessed health parameters (diarrhea or pneumonia). It is known that diarrhea and respiratory problems are caused by a combination of factors and related to the immunity status, nutrition, type of housing, and season [19]. In accordance with those findings, research conducted with calves fed with BTM, WM, or PWM reported no differences in fecal score [1]. In another study, researchers observed that calves fed WM containing antibiotic residues showed fewer diarrhea symptoms than calves fed milk replacer; however, no direct relationship between the antibiotic residues and such an effect was reported [30]. The effects of antibiotic residues on diarrhea are small [31,32]. In our experiment, the lack of differences in the health parameters between treatments is possibly due to the intense cleaning of facilities and utensils used during animal handling, which may have reduced animal contamination and disease transmission. However, a retrospective analysis using data from the study revealed that the experimental power was 70% for fecal score, which limits the inferential capacity of the variable.

The similar values in the performance parameters (final BW, total weight gain, and ADG) were due to the similar DMI in all treatments. The ADG values were affected only by week, with lower ADG at the second week (0.44 kg/d), coinciding with the period of higher incidence of diarrhea (15 d ± 6.6) and higher fecal score (0.81). The week factor significantly affected the body measurement parameters, with steady increases of WH, RH, RW, and HG at each week. In addition, a study with calves receiving WM, PWM, BTM, or pasteurized whole milk also did not observe differences in ADG values from 0–14, 15–28, or 29–56 d [25]. On the other hand, a previous study reported higher ADG in calves receiving WM and PWM in comparison to calves fed BTM [1]. However, the experimental period was shorter than our experiment (21 d), making direct comparisons with our data difficult.

The differences in experimental design make comparisons and conclusions regarding the effects of feeding WM or PWM on the health and performance of calves challenging. In general, the scientific literature still offers controversial results regarding the usage of WM. Due to the contribution of colostrum and transition milk, WM may present higher concentrations of total solids. In turn, higher solids can contribute to health and performance gains, masking the negative effects of microbial contamination and antibiotic residues. Regarding pasteurized WM, flaws in the pasteurization process that jeopardize its efficiency, possible spoilage of milk content, and milk recontamination are factors that may impair the beneficial effects of PWM on the health and performance of animals.

## 5. Conclusions

Feeding waste milk (WM) and pasteurized WM did not show significant negative effects on the intake, ruminal parameters, blood parameters, health, or performance of crossbred dairy calves.

## Figures and Tables

**Table 1:** Composition, somatic cell count (SCC), and total bacterial count (TBC) of bulk tank milk (BTM), waste milk (WM), and pasteurized waste milk (PWM) samples and composition of concentrate used during the period from 4 to 60 d.

Item	Treatment *^1^			SEM	Starter ^2^
	BTM	WM	PWM		
**Composition (%)**					
DM	13.04 (0.58) a	12.82 (0.64) b	12.48 (0.53) c	0.51	94.53
Fat	4.24 (0.54) a	4.10 (0.58) a	3.76 (0.43) b	0.46	3.14
CP	3.30 (0.27) b	3.46 (0.44) a	3.49 (0.29) a	0.24	19.06
Casein	2.71 (0.19)	2.64 (0.36)	2.53 (0.44)	0.04	-
Non-protein nitrogen	0.10 (0.01) b	0.13 (0.02) a	0.12 (0.02) a	<0.01	-
Lactose	4.46 (0.12) a	4.33 (0.20) b	4.33 (0.17) b	0.15	-
Ash	0.69 (0.03) b	0.72 (0.04) a	0.73 (0.03) a	<0.01	8.81
NDF	-	-	-	-	12.70
ADF	-	-	-	-	5.60
GE (Kcal/kg)	-	-	-	-	4168.63
**Milk Quality**					
SCC (×10^3^ cells/mL)	366.81 (175.13) c	1740.15 (1638.03) a	1424.67 (784.44) b	901.00	-
TBC (×10^3^ UFC/mL)	19.79 (15.14) c	548.37 (695.11) a	295.41 (353.25) b	369.00	-

* Values in parentheses indicate standard deviation of the values of each treatment. ^1^ Means followed by a lowercase letter represent statistical difference between treatments (*p* < 0.05; Tukey test). ^2^ Basic composition: soybean meal, ground corn, and mineral (Prima/DSM, São Paulo, Brazil).

**Table 2:** Intake, performance, feed efficiency, and body measurements of dairy calves fed bulk tank milk (BTM, *n* = 15), waste milk (WM, *n* = 15), and pasteurized waste milk (PWM, *n* = 15) during the period from 4 to 60 d of age.

Item	Treatment ^6^			SEM	*p*-Value ^1^		
	BTM	WM	PWM		T	W	T × W
**Intake**							
Milk (g of DM/d)	772.37 a	764.29 b	740.87 c	1.20	<0.01	0.06	0.69
Starter (g of DM/d)	129.95	159.72	162.44	0.73	0.13	<0.01	<0.01
Total DMI ^2^ (g of DM/d)	895.34	911.17	899.98	11.81	0.93	<0.01	0.64
Total gross energy ^3^ (Mcal/d)	3.48	3.91	4.23	1.21	0.21	<0.01	<0.01
Total CP ^4^ (kg/d)	50.75	57.35	59.22	5.19	0.27	<0.01	<0.01
Water (L/d)	1.87	1.84	1.86	0.08	0.95	<0.01	0.09
**Performance**							
Initial weight (kg)	38.49	38.19	39.51	0.43	0.62	-	-
Final weight (kg)	76.03	77.43	74.09	0.82	0.77	-	-
Average of total period (kg)	37.55	39.25	34.58	0.36	0.13	-	-
ADG (kg/d)	0.67	0.71	0.62	0.03	0.25	<0.01	0.26
**Feed efficiency ^5^**	0.76 a	0.79 a	0.71 b	0.03	<0.01	<0.01	<0.01
**Body measures (cm)**							
Heart girth	86.94	86.16	86.23	1.33	0.21	<0.01	<0.01
Withers height	84.41	84.15	84.28	0.79	0.74	<0.01	0.73
Hip width	24.95	25.01	24.82	0.56	0.35	<0.01	0.75
Hip height	87.19	87.35	87.36	0.92	0.88	<0.01	0.06

^1^ T = treatment effect; W = week effect; T × W = treatment × week interaction; ^2^ Total DMI = starter DM + milk DM intakes; ^3^ Total gross energy = starter GE + milk GE intakes; ^4^ Total CP = starter CP + milk CP intakes; ^5^ feed efficiency was calculated by dividing ADG (g) by average daily DMI; ^6^ Means followed by a lowercase letter represent statistical difference between treatments (*p* < 0.05; Tukey test).

**Table 3:** Ruminal and blood parameters of dairy calves fed bulk tank milk (BTM, *n* = 15), waste milk (WM, *n* = 15), and pasteurized waste milk (PWM, *n* = 15) during 4–60 d of age.

Item	Treatment ^1^			SEM	*p*–Value ^2^		
	BTM	WM	PWM		T	W	T × W
**Rumen parameters**							
pH	5.81	5.64	5.58	0.19	0.10	<0.01	0.84
Ammonia-N (mg/dL)	17.71	19.02	21.18	0.08	0.36	0.48	0.35
Volatile Fatty Acids (mmol/L)							
Acetic (C2)	33.02 b	32.89 b	38.48 a	8.33	0.02	<0.01	0.50
Propionic (C3)	21.68 b	21.22 b	26.21 a	0.71	0.03	<0.01	0.41
Butyric (C4)	4.64	5.49	6.13	0.02	0.08	0.07	0.26
C2:C3	1.57	1.52	1.50	0.01	0.49	<0.01	0.86
**Blood parameters**							
Glucose (mg/dL)	97.36	102.14	94.11	24.51	0.33	0.19	0.75
BHB (mmol/dL)	0.13	0.12	0.11	0.01	0.68	<0.01	0.31

^1^ Means followed by a lowercase letter represent statistical difference between treatments (*p* < 0.05; Tukey test); ^2^ T = treatment effect; W = week effect; T × W = treatment × week interaction.

**Table 4:** Health parameters of dairy calves fed bulk tank milk (BTM, *n* = 15), waste milk (WM, *n* = 15), and pasteurized waste milk (PWM, *n* = 15) during the period from 4 to 60 d of age.

Item	Treatment			SEM	*p*–Value ^1^		
	BTM	WM	PWM		T	W	T × W
**Diarrhea**							
Fecal score ^2^	0.65	0.57	0.51	0.08	0.58	<0.01	0.08
Days with diarrhea (d)	8.21	5.93	5.21	1.44	0.29	-	-
Days with fever (d)	0.67	1.13	1.81	0.94	0.79	-	-
**Pneumonia**							
Pulmonary consolidation ≥1 cm (*n*) ^3^	3	7	5	-	0.3	-	-

^1^ T = treatment effect; W = week effect; T × W = treatment × week interaction; ^2^ Fecal score: 0, normal (firm but not hard); 1, soft (does not hold form, piles but spreads slightly); 2, runny (spreads readily to about 6-mm depth); and 3, watery (liquid consistency, splatters) [18]; ^3^ number of animals with lung consolidation ≥1 cm.

## Data Availability

The data that support the findings and that are presented in this study are available on reasonable request from the corresponding author, Mariana Magalhães Campos, mariana.campos@embrapa.br. The data are not publicly available because not all of the study’s data have been published yet.
